# Diet Quality Among Mothers and Children in India: Roles of Social and Behavior Change Communication and Nutrition-Sensitive Social Protection Programs

**DOI:** 10.1016/j.tjnut.2024.07.026

**Published:** 2024-07-23

**Authors:** Phuong Hong Nguyen, Sumanta Neupane, Anjali Pant, Rasmi Avula, Anna Herforth

**Affiliations:** 1 Nutrition, Diets, and Health Unit, International Food Policy Research Institute, Washington, DC, United States; 2 Global Health and Population, Harvard T.H. Chan School of Public Health, Boston, MA, United States

**Keywords:** Diet Quality Questionnaire (DQQ), social and behavior change communication (SBCC), nutrition-sensitive social protection (NSSP), India

## Abstract

**Background:**

Limited evidence exists on determinants of maternal and child diet quality.

**Objectives:**

This study examined the role of social and behavior change communication (SBCC) and nutrition-sensitive social protection (NSSP) programs on maternal and child diet quality.

**Methods:**

Data were from cross-sectional phone survey on 6627 Indian mothers that took place in late 2021. The Diet Quality Questionnaire (DQQ) was used to measure maternal and child diet quality, including minimum dietary diversity (MDD), dietary diversity scores (DDSs), noncommunicable disease (NCD)-protect and NCD-risk scores, adherence to dietary guidelines (All-5 and India-All-6), and unhealthy child feeding. Multivariate regression models were used to explore the association between diet indicators and coverage of SBCC and NSSP programs.

**Results:**

Maternal and child diet quality was suboptimal, with more mothers (57%) achieving MDD than children (23%). SBCC was positively associated with healthy food consumption in children (odds ratio [OR]: 2.14 for MDD; β: 0.60 for DDS and 0.54 for NCD-protect) and mothers (β: 0.38 for DDS and 0.43 for NCD-protect). Cash transfers were associated with healthier diets in mothers (OR: 1.45 for MDD, 1.86 for All-5, and 2.14 for India-All-6; β: 0.43 for DDS and 0.26 for NCD-protect), but less associations noted for children (β: 0.14 for NCD-protect). Receiving food was associated not only with healthier diets in mothers (OR: 1.47 for MDD; β: 0.27 for DDS and 0.33 for NCD-protect) and children (β: 0.19 for DDS and 0.15 for NCD-protect) but also with unhealthy food in children (OR: 1.34). Exposure to multiple programs showed stronger associations with diet quality.

**Conclusions:**

SBCC has greater positive impact on child feeding than food and cash transfers, while cash has a stronger association with improved maternal diets. Food and cash are also associated with unhealthy food consumption. Our study underscores the importance of interventions that combine education, resource provision, and targeted support to promote maternal and child diet quality.

## Introduction

Suboptimal diets have been identified as a major cause of mortality and morbidity worldwide. Globally, in 2017, diet-related risk factors for noncommunicable diseases (NCDs) were responsible for 11 million deaths (22%) and 255 million disability-adjusted life years (15%), surpassing the impact of other risk factors [1]. The role of diet is particularly critical during pregnancy, lactation, and early childhood because it is an important contributor to maternal health and it lays the foundation for health, cognition and productivity of future generations [2]. Yet, maternal and child diets in many low-income and middle-income countries (LMICs) are primarily reliant on starchy staple foods with imbalanced macronutrients and inadequate micronutrient intakes [3]. Poor maternal and child diets became more severe during COVID-19 pandemic when food insecurity and hunger disproportionately affected South Asia [[4], [5], [6]].

Large-scale social and behavior change communication (SBCC) programs implemented across several countries demonstrate that exposure to multiple intervention channels, such as interpersonal communication, community mobilization, mass media, was associated with improvements in maternal diet diversity [[7], [8], [9]] and infant and young child feeding (IYCF) practices [9,10]. Evaluations of nutrition-sensitive social protection (NSSP) programs, such as food supplementation and cash transfer programs, indicate that they improve the affordability of diets [11] and dietary diversity [11,12]. NSSP programs were even more relevant during the COVID-19 pandemic when strict lockdown measures posed significant risks to household food security, maternal diets, and child feeding practices [13,14].

India, the world’s most populous country with a population exceeding 1 billion, faces a significant challenge, with an estimated 63%–76% of its population unable to afford a healthy diet [15,16]. In 2019–2021, only 13% of women reported consuming fruits daily; about half consumed vegetables, pulses, and dairy products; and <10% consumed other animal-source foods [17]. Child dietary diversity is also stagnantly low, with only 24% of children aged 6–23 months achieving minimum dietary diversity (MDD) in 2019–2021 [18].

To address challenges of suboptimal diets in India, a range of policies and programs are currently underway. These initiatives include SBCC programs, social protection strategies and safety nets (i.e. food-based programs or cash transfers), distribution of micronutrient supplements, promotion of food fortification, and nutrition-sensitive agricultural activities [19]. India has implemented numerous NSSP programs aimed at tackling food insecurity and improving nutrition, particularly focusing on the first 1000 days and targeting women during pregnancy or lactation, as well as young children. Key programs include the following: *1*) the Public Distribution System, which provides subsidized food grains, pulses, and oil to eligible beneficiaries; *2*) the Integrated Child Development Services (ICDS), which offer nutritional counseling and tailored food supplements like Take Home Ration or Hot Cooked Meals, in the form of micronutrient fortified food and/or energy-dense food, to promote the nutritional well-being of pregnant and lactating women, as well as young children; and *3*) the maternity benefits program, which provides cash transfers to pregnant women who adhere to health care protocols during pregnancy, delivery, and postpartum periods [20,21]. In response to the COVID-19 pandemic, the government introduced an additional food transfer scheme to mitigate income loss resulting from mobility restrictions.

Despite the wide spread of NSSP programs, little is known how they influence maternal and child diet, mainly because of challenges related to collecting diet data such as high cost, time burden, complexity, and limited technical capacity [22]. Previous research examining NSSPs found significant impacts of cash transfer programs on maternal and child health outcomes [23,24], as well as institutional deliveries rates [24]. However, there is limited understanding if such programs could influence dietary quality. Among various methods available, the Diet Quality Questionnaire (DQQ) is a standardized low burden tool for dietary assessment at the population level [25,26]. Using this questionnaire, maternal dietary diversity and other diet quality indicators can be calculated, and a companion questionnaire is used for assessing IYCF indicators. This study used the DQQ to assess diet quality for both mothers and their children in a programmatic context and to examine the association between diet quality and participation in social protection programs. The study had 2 main objectives: *1*) to examine maternal and child diet quality, in terms of both healthy and unhealthy food consumption, in India and *2*) to assess individuals and combined associations of SBBC and NSSP programs on maternal and child diet quality.

## Methods

### Study context

This study was part of larger study that assessed disruptions and restorations of health and nutrition service delivery and utilization during the COVID-19 pandemic in India [27]. The initial phase of the study, conducted between July and October 2020, focused on frontline workers with the aim of understanding maternal and child health service delivery amidst the COVID-19 pandemic. The subsequent phase, carried out between October and December 2021, targeted households to understand their access to and utilization of various essential services such as social safety net programs and health and nutrition services. We conducted a phone survey with 6627 mothers with children younger <2 y in the states of Chhattisgarh, Gujarat, Madhya Pradesh, Odisha, Telangana, and Uttar Pradesh.

The states were selected to align with existing data collection opportunities within ongoing studies or existing research collaborations with state governments. In each state, we randomly selected 3 rural districts, and within each district, we then randomly chose 20 villages. To identify eligible households (those with mothers having children younger than 2 y), we gathered phone numbers from a registration list provided by village health workers. Within each village, we used a systematic random sampling method to select 15–17 respondents. Under this method, samples were selected at intervals calculated as the ratio of the total eligible samples to the required samples, with the first sample being drawn randomly. Subsequently, our team reached out to these women, articulating the study’s purpose, their rights and responsibilities, and the confidentiality procedures through a consent form read out in the local language. In case of refusals, we replaced the respondent by the next women in the sampling frame. The final response rate was high, ranging from 85% in Madhya Pradesh to 98% in Chhattisgarh and Telangana.

At the outset of the phone survey, we obtained oral consent from all participants. Ethical approval for the study was granted by the institutional review board from the respective institutions involved (IRB reference number: PHND-20-0723TMM) and approval from state governments.

### Diet quality measures

Maternal diet was measured using the DQQ that gathers food group consumption data required for calculating diet quality indicators, comprising a set of yes/no questions ∼29 food groups consumed in the previous day or night [26]. This questionnaire was validated and implemented in 85 countries in the Gallup World Poll in 2021–2023 [25,26,28] and was adapted for Indian-specific context [29]. For children, we used the DQQ version adapted for IYCF. Data from DQQ were used to compute MDD and dietary diversity score (DDS), foods to consume in moderation (NCD-risk score), and foods that can help prevent NCDs (NCD-protect score). These indicators are separately calculated for women and children.

Among women, the DDS was calculated by counting the food groups consumed across the following 10 food groups: *1*) grain, roots, tubers, and plantains; *2*) pulses; *3*) nut and seeds; *4*) dairy; *5*) meat; *6*) poultry, fish, and eggs; *7*) dark green leafy vegetables; *8*) vitamin A–rich fruits and vegetables; *9*) other vegetables; and *10*) other fruits. The MDD for women was defined as consumption of 5 of 10 food groups [30]. Among children, DDS was the sum of the following 8 food groups: *1*) breastmilk; *2*) grains, roots, tubers, and plantains; *3*) pulses, nuts, and seeds; *4*) dairy; *5*) meat fish, poultry, and organ-meat; *6*) eggs; *7*) vitamin A–rich fruits and vegetables; and *8*) other fruits and vegetables. MDD for children was defined as consumption of 5 of 8 food groups [31].

The NCD-protect score reflects consumption of a variety of plant-based foods associated with lower risk of NCDs. It is based on the following 9 food groups: *1*) whole grains; *2*) pulses; *3*) nuts and seeds; *4*) vitamin A–rich orange vegetables; *5*) dark green leafy vegetables; *6*) other vegetables; *7*) vitamin A–rich fruits; *8*) citrus; and *9*) other fruits. The NCD-protect score is calculated by counting the food groups consumed with a higher score indicating more health-promoting foods consumption [25].

The NCD-risk score assesses consumption of foods high in sugar, salt, fat, and processed meat, associated with higher risk of NCDs [32]. It consists of the following 8 food groups: *1*) soft drinks; *2*) grain-based sweets; *3*) other sweets; *4*) processed meat; *5*) unprocessed red meat; *6*) deep fried food; *7*) fast food and instant noodles; and *8*) packaged ultraprocessed salty snacks. The NCD-risk score is calculated by counting the food groups consumed (double weighting processed meat); a higher score indicates higher consumption of foods and drinks to avoid or limit and correlates negatively with meeting global dietary recommendations for the general population [25]. This study modified the NCD-risk score for children (range: 0–7) as a count of unhealthy food consumption, including the following 6 food groups: *1*) soft drinks; *2*) fruit juice; *3*) sweetened tea/coffee; *4*) sweets; *5*) processed meats (double weighting); and *6*) fried food, noodles, and processed snacks. NCD-risk factor for young children differs from adults in that it does not include unprocessed red meat, adds 2 additional types of sweetened beverages not recommended for IYC, and groups unhealthy sweet foods and unhealthy salty snacks together. These modifications to the score for IYC were made to better align with the IYCF unhealthy food and sweet beverage indicators published by WHO and UNICEF 2021 [31].

In addition, we calculated the “All-5” indicator of consuming 5 globally recommended food groups (≥1 vegetable, 1 fruit, 1 pulse, nut, or seed, 1 animal-source food, and 1 starchy staple) [32]. We also calculated a modified form of this indicator (India-All-6) to reflect the 6 food groups in India’s national food-based dietary guidelines (≥1 each of cereals, pulses or eggs or fish or meat, milk or curd, vegetables, fruits, and nuts or seeds) as recommended by the National Institute of Nutrition [33]. These indicators are more relevant for adults, because they refer to food-based dietary guidelines that generally are formulated for the general population older than 2 y. For children aged 6–23 mo, we calculated 3 new IYCF indicators: zero vegetable or fruit consumption, unhealthy food consumption, and sweet beverage consumption [31].

### Social protection and nutrition counseling program participation

Our main explanatory variables were NSSP interventions as well as health and nutrition counseling received during pregnancy, lactation, and for the child. Under the ICDS program, all pregnant women in India are eligible to receive food supplements during pregnancy. Women who are pregnant for the first time are eligible to receive cash during pregnancy under the maternal benefit program, Pradhan Mantri Matru Vandana Yojana. Any child aged 6–59 mo is eligible for food supplementation under ICDS, and the child’s mother is also eligible for cash after completion of first round of vaccination under the Pradhan Mantri Matru Vandana Yojana; however, this cash transfer is only applicable for the first child. Under the ICDS and the health programs, women are expected to receive health and nutrition counseling during pregnancy and breastfeeding and IYCF counseling during lactation.

Exposure to NSSP programs and counseling were assessed by directly asking women using a survey questionnaire (Supplemental Questionnaire), which was carefully designed and tested using cognitive testing methods as well [34]. Specifically, respondents were asked if they had received cash during their last pregnancy or for delivery at a health facility, as well as whether they had received food supplements during pregnancy. Additionally, we asked whether they had received counseling on health and nutrition from health care providers during pregnancy. Similarly, for their youngest child, respondents were asked if they had received food assistance, cash after completing child vaccination, and counseling on child feeding practices within the past 2 y.

We examined the relationships between diet quality and exposure to either individual programs or combination of multiple programs (food supplementation, cash transfers, and counseling). Given that women or children could be exposed to several programs simultaneously, they were categorized into 4 exposure groups: *1*) not exposed to any NSSP or SBCC; *2*) exposed to only 1 NSSP or SBCC; (3) exposed to 2 NSSPs or SBCC; and (4) exposed to all 3 programs.

### Covariates

We included both individual-level and household-levels covariates such as child’s age (months), mother’s age (years), education (number of years of schooling completed), occupation (home maker or employed), and caste (scheduled caste or tribe, backward class, or other caste). Household wealth index was constructed using principal component analysis of 22 household assets [35] and was then standardized to a scale ranging from 0 to 10.

### Statistical analysis

We calculated frequencies for the diet quality indicators (DDS, MDD, NCD-protect, NCD-risk, All-5, India-All-6, zero vegetable or fruit consumption, unhealthy food consumption, and sweet beverage consumption) and the 29 food groups that contributed to these indicators. To examine the association between diet indicators and coverage of cash and food and health nutrition counseling, we used ordinary least square regressions for continuous outcomes (DDS, NCD-protect, and NCD-risk), and logistic regressions for binary outcomes (for mothers: MDD, All-5, India-All-6, and for children: MDD, zero vegetable or fruit consumption, unhealthy food consumption, and sweet beverage consumption). To account for benefits received during pregnancy and by the child while the mother was lactating, we performed separate regressions for mothers and children. All regressions were adjusted for wealth, caste, mother’s education, occupation, mother’s age, and child’s age. We included state fixed effects to control for unmeasured factors at the state level. We reported either β coefficient or odds ratio (OR) and CIs and used significance levels at ∗*P* < 0.05, ∗∗ *P* < 0.01, and ∗∗∗ *P* < 0.001.

## Results

### Characteristics of the study population

Table 1 provides an overview of the demographic, socioeconomic, and social protection characteristics of the surveyed population. On average, mothers were aged ∼25 y and their children ∼1 y. Nearly half of mothers had ≥10 y of schooling, but 87% were housewives. Compared with the sample from the 2019–2021 India National Family Health survey, our sample had higher proportion of mothers with ≥10 y of schooling, except in Gujarat (Supplemental Table 1). Additionally, the age distribution of mothers differed between the 2 surveys, with our sample being younger, except in Uttar Pradesh.

**TABLE 1:** Sample characteristics^1^

	N	% or mean ± SD
Mother’s age (y)	6627	25.4 ± 3.7
Child’s age (mo)	6627	11.5 ± 7.0
Mother’s education (%)		
No schooling	476	7.2
<5 y	278	4.2
5–9 y	2575	38.9
10–11 y	1393	21.0
12 y or more	1905	28.7
Occupation (%)		
Employed	884	13.3
Homemaker	5738	86.7
Caste (%)		
Scheduled caste or tribe	2359	35.8
Other backward classes	3412	51.8
Other caste categories	821	12.5
Household wealth index (score 0–10)	6627	5.4 ± 1.3
Social protection scheme—mothers (%)		
Received food from government during last pregnancy	6565	88.2
Received cash from government during last pregnancy	4701	60.4
Health and nutrition counseling during pregnancy	6563	94.1
Social protection scheme—children (%)		
Received food from government for youngest child	4720	72.2
Received cash from government for youngest child	5063	42.9
Counseling on infant and young child feeding	4720	83.5

Abbreviation: SD, standard deviation.

1 Values are means ± SDs or percentages.

A significant proportion of mothers received food (88.2%) and cash transfers (60.4%) during their last pregnancy. During lactation, 72.2% received food, and 42.9% received cash transfers for the youngest child. A majority of mothers (94.1%) received health and nutrition counseling during pregnancy, and 83.5% received counseling on IYCF. Among mothers, a majority (58.8%) received all 3 interventions, 31.7% received ≥2 interventions (31.7%), while 6.7% received only 1 intervention, and 2.8% did not receive any intervention. Among children, patterns of combined intervention exposure differed, with a smaller percentage (10.9%) receiving all 3 interventions, 60.4% receiving ≥2 interventions, 22.6% receiving only 1 intervention, while 6.1% did not receive any intervention.

### Food group consumption

**FIGURE 1:**
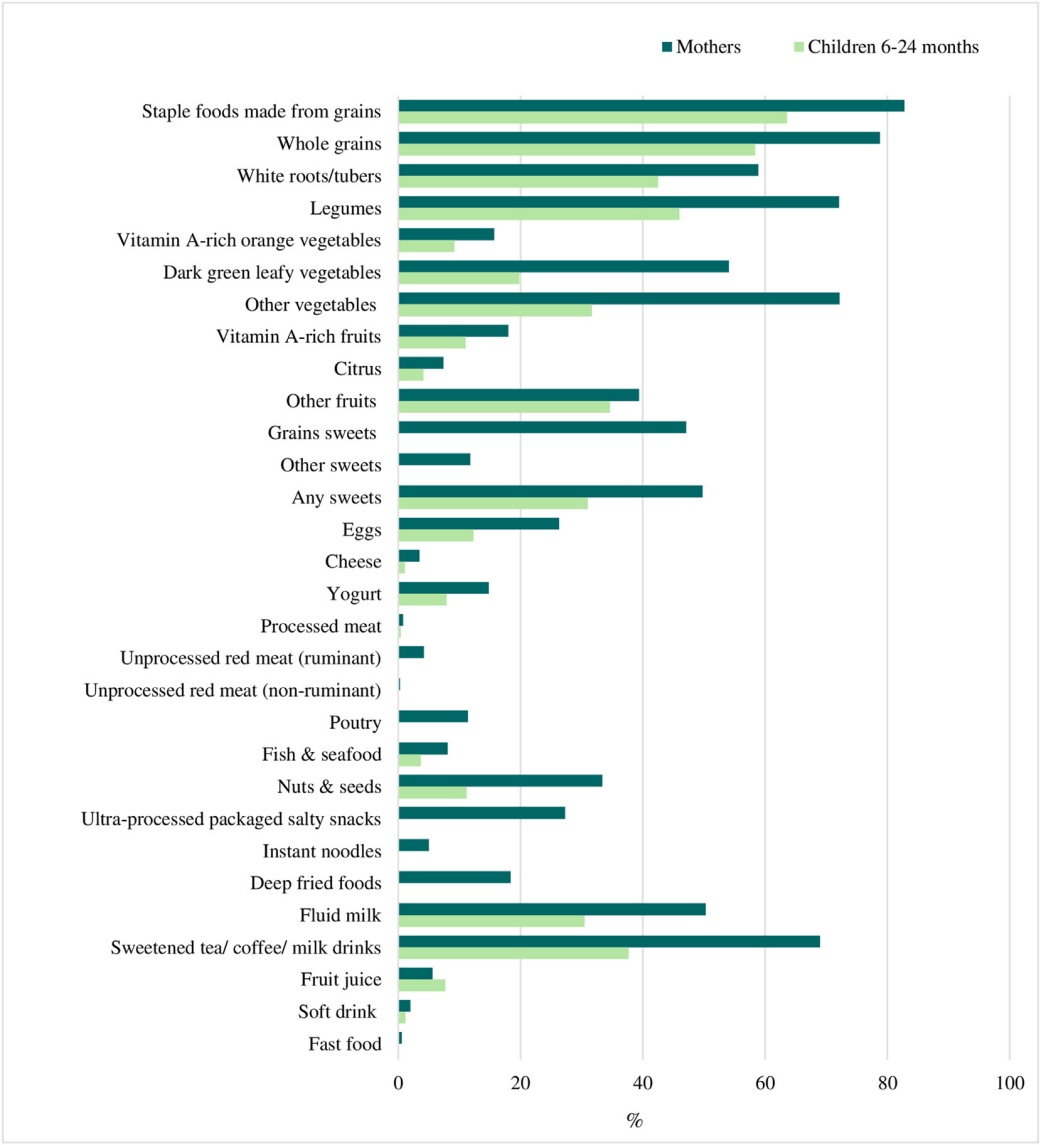
Prevalence of consumption various food groups among mothers and children^1^. ^1^Information on consumption of grain sweets, other sweets, unprocessed meat (ruminant), unprocessed meat (nonruminant), poultry, ultraprocessed packaged salty snacks, instant noodles, deep fried food, and fast food is not available for children, because these food groups were collapsed into larger umbrella food groups (e.g., sweet foods, flesh foods, savory unhealthy foods).

Food group consumption, as measured by the DQQ for mothers and children, is depicted in Figure 1 and Supplemental Table 2. As expected, starchy staple foods were commonly consumed by both mothers (83%) and children (64%), whereas animal-source foods (including dairy, eggs, meat, poultry, fish, and sea food) were less frequently consumed by mothers (68%) and children (46%). Although more than half of mothers included dark green leafy vegetables (54%) and other vegetables (72%) in their diet, a smaller percentage of mothers consumed vitamin A–rich orange vegetables (16%) and vitamin A–rich fruits (18%). In case of unhealthy foods, 69% of mothers consumed sweetened tea, coffee, or milk drinks, 50% consumed sweet foods, 27% consumed ultraprocessed packaged salty snacks, and 18% consumed deep fried foods. Overall, children exhibited lower consumption across all food groups than mothers, except for fruit juice (consumed by 8% of children).

### Diet quality indicators

The mean maternal DDS was 5.0 ± 1.9 (of 10 food groups), while the child DDS was 3.8 ± 1.7 (of 8 food groups) (Table 2). Notably, more than half of the mothers (57%) achieved an MDD compared with 23% of children. Details of food groups contributing to DDS for mothers and children are presented in Supplemental Table 2.

**TABLE 2:** Diet quality indicators among mothers and children

Indicators	Mother (*n* = 6627)	Children, 6–23 mo (*n* = 4865)
Dietary Diversity Score (mean ± SD)^1^	5.0 ± 1.9	3.8 ± 1.7
Minimum dietary diversity (%)	57.0	23.3
NCD-protect score (mean ± SD)	3.9 ± 1.7	2.3 ± 1.8
NCD-risk score (mean ± SD)^2^	1.2 ± 1.3	1.0 ± 1.0
Consumed all-5 food groups recommended across countries globally^3^ (%)	29.8	—
Consumed all-6 food groups recommended in India^4^ (%)	12.2	—
Zero vegetable or fruit consumption, 6–23 mo (%)	—	41.7
Unhealthy food consumption, 6–23 mo (%)	—	37.7
Sweet beverage consumption, 6–23 mo (%)	—	48.8

Abbreviation: NCD, noncommunicable disease; SD, standard deviation.

1 Minimum dietary diversity for women is defined as consuming ≥5 of 10 food groups (FAO, 2021), while minimum dietary diversity for children is defined as consuming ≥5 of 8 food groups (WHO and UNICEF, 2021).

2 NCD-risk score is defined as mean score of consumption of 8 food groups to limit that may increase mother’s vulnerability to NCDs, with a range of 0–9 (processed meat is double weighted). NCD-risk score modified for young children (range: 0–7) is the mean score of consumption of 6 food unhealthy groups for young children, which differs from the adult score in the following ways: it does not include unprocessed red meat, adds 2 additional types of sweetened beverages not recommended for IYC, and groups unhealthy sweet foods and unhealthy salty snacks together. These modifications to the score for IYC were made to better align with the IYCF unhealthy food and sweet beverage indicators published by WHO and UNICEF, 2021.

3 The 5 food groups are starchy staples; vegetables; fruits; pulses, nuts or seeds; animal-source foods.

4 The 6 food groups are cereal grains; vegetables; fruits; nuts or seeds; pulses, eggs, fish, or meat; dairy.

The NCD-protect score was higher among mothers than that among children (3.9 ± 1.7 compared with 2.3 ± 1.8). The NCD-risk score was low among both mothers (1.2 ± 1.3 of 9 food groups) and children (1.0 ± 1.0 of 7 food groups). Nearly 30% of mothers reported consuming all 5 globally recommended food groups, whereas adherence to the 6 food groups recommended in India was notably lower, with only 12% of mothers meeting these dietary guidelines for daily consumption (Table 2). Among the food groups recommended in India-All-6, the most commonly lacking food group is nut and seeds (absent in 67% of mothers’ diets), followed by fruits (missing in 51% of diets), and milk or curd (missing in 46% of diets) (Supplemental Table 2). Other indicators of IYCF for children were also concerning, with 42% consuming zero vegetable or fruit consumption, 38% consuming unhealthy foods, and nearly half (49%) consuming sweet beverages (Table 2), predominantly sweetened tea or milk drinks.

### Association between diet quality indicators and NSSP programs and SBCC counseling among mothers

Exposure to cash transfers, food supplementation, and counseling on health and nutrition (either individually or in combination) was associated with diet quality among mothers (Table 3). Specifically, exposure to only health and nutrition counseling during pregnancy had a significant positive relationship with DDS (β: 0.38; 95% CI: 0.10, 0.65) and NCD-protect (β: 0.43; 95% CI: 0.29, 0.57). However, no significant relationship was observed with NCD-risk. Similarly, receiving food supplementation during pregnancy was associated with higher DDS (β: 0.27; 95% CI: 0.06, 0.47), higher odds of achieving MDD-W (OR: 1.47; 95% CI: 1.16, 1.87), along with a higher NCD-protect score (β: 0.33; 95% CI: 0.14, 0.51), but not with NCD-risk. Women who received cash transfers during their last pregnancy had better diet quality than those who did not, including approximately double the odds of consuming All-5 food groups based on global recommendations and India-All-6 food groups recommended for India (OR: 1.86 for All-5 and 2.14 for All-6).

**TABLE 3:** Association between health and nutrition counselling, social protection program, and diet quality indicators among mothers^1^

	MDD^2^	Dietary Diversity Score	NCD-protect score	NCD-risk score^3^	All-5 food groups^4^, global recommended	All 6 food groups^5^, recommended for India
	OR (95% CI)	β (95% CI)	β (95% CI)	β (95% CI)	OR (95% CI)	OR (95% CI)
Exposure to individual interventions during pregnancy						
Received health and nutrition counseling	1.35 (0.98, 1.86)	0.38∗ (0.10, 0.65)	0.43∗∗∗ (0.18, 0.68)	−0.08 (−0.27, 0.10)	1.06 (0.74, 1.52)	1.23 (0.74, 2.03)
Received food from government	1.47∗∗ (1.16, 1.87)	0.27∗ (0.06, 0.47)	0.33∗∗∗ (0.14, 0.51)	0.09 (−0.05, 0.22)	1.22 (0.93, 1.60)	1.35 (0.93, 1.95)
Received cash from government	1.45∗∗∗ (1.23, 1.70)	0.43∗∗∗ (0.29, 0.57)	0.26∗∗∗ (0.13, 0.38)	0.10∗ (0.01, 0.19)	1.86∗∗∗ (1.57, 2.21)	2.14∗∗∗ (1.69, 2.72)
Exposure to 1 or more interventions during pregnancy						
Received only 1 intervention	1.07 (0.69, 1.66)	0.21 (−0.17, 0.59)	0.33^+^ (−0.01, 0.68)	−0.21 (−0.46, 0.04)	0.93 (0.56, 1.55)	0.89 (0.45, 1.77)
Received ≥2 interventions	1.59∗ (1.07, 2.35)	0.47∗∗ (0.14, 0.81)	0.64∗∗∗ (0.33, 0.95)	−0.12 (−0.35, 0.10)	1.04 (0.66, 1.64)	1.18 (0.64, 2.16)
Received all 3 interventions	2.40∗∗∗ (1.61, 3.57)	0.94∗∗∗ (0.60, 1.28)	0.95∗∗∗ (0.64, 1.26)	−0.01 (−0.24, 0.22)	1.99∗∗ (1.26, 3.13)	2.52∗∗ (1.38, 4.61)

Abbreviations: CI, confidence interval; MDD, minimum dietary diversity; NCD, noncommunicable disease; OR, odds ratio.

+*P* < 0.10; ∗*P* < 0.05; ∗∗*P* < 0.01; ∗∗∗*P* < 0.001.

1 All models were controlled for covariates including wealth, caste, women education, women employment status, women age, child age, and state fixed effects. 95% CI in parenthesis. *N* = 4817 for the mothers’ models.

2 Minimum dietary diversity for women is defined as consuming ≥5 of 10 food groups (FAO, 2021).

3 NCD-risk score is defined as mean score of consumption of 8 food groups to limit that may increase mother’s vulnerability to NCDs, with a range of 0–9 (processed meat is double weighted).

4 In the all-5 indicator, the 5 food groups are starchy staples; vegetables; fruits; pulses, nuts or seeds; animal-source foods.

5 The 6 food groups are cereal grains; vegetables; fruits; nuts or seeds; pulses, eggs, fish, or meat; dairy.

Women who were exposed to multiple programs had stronger association with diet quality indicators than those exposed to fewer programs. For instance, compared with women who were not exposed to any programs, those exposed to all 3 programs were 2.4 times more likely to have achieved MDD-W. This association decreased to 1.6 times among those exposed to 2 programs, with no significant difference observed among those exposed to only 1 program. Similar dose–response relationships were evident between combined exposure and other dietary indicators such as DDS, NCD-protect, consumption of All-5 food groups, and India-all-6 food groups (Table 3).

### Association between diet quality indicators and NSSP programs and SBCC counseling and among children

Exposure to cash transfers, food supplementation, or IYCF counseling, or any combination of these programs, was associated with diet quality among children (Table 4). Specifically, IYCF counseling demonstrated a significant positive relationship with DDS (β: 0.60; 95% CI: 0.44, 0.75), NCD-protect score (β: 0.54; 95% CI: 0.38, 0.71), and was associated with double the odds of achieving MDD (OR: 2.14; 95% CI: 1.65, 2.78), and half the odds of consuming zero vegetables or fruits (OR: 0.48; 95% CI: 0.39, 0.60). Additionally, counseling also had a small but significant association with NCD-risk score (β: 0.13; 95% CI: 0.02, 0.23). Receiving food from the government for the youngest child had significant correlations of small magnitude not only with greater child DDS (β: 0.19; 95% CI: 0.06, 0.32) and NCD-protect score (β: 0.15; 95% CI: 0.01, 0.30) but also with NCD-risk score (β: 0.14; 95% CI: 0.05, 0.23) and unhealthy food consumption (OR: 1.34; 95% CI: 1.10, 1.63) (Table 4). Finally, receiving cash transfers from the government was only associated with NCD-protect score (β: 0.14; 95% CI: 0.02, 0.26).

**TABLE 4:** Association between health and nutrition counseling, social protection program, and diet quality indicators among children (12–24 mo)^1^

	MDD^2^	Dietary Diversity Score	NCD-protect score	NCD-risk score^3^	Zero vegetable or fruit consumption	Unhealthy food consumption	Sweet beverage consumption
	OR (95% CI)	β (95% CI)	β (95% CI)	β (95% CI)	OR (95% CI)	OR (95% CI)	OR (95% CI)
Exposure to individual interventions during lactation or for children							
Received IYCF counseling	2.14∗∗∗ (1.65, 2.78)	0.60∗∗∗ (0.44, 0.75)	0.54∗∗∗ (0.38, 0.71)	0.13∗ (0.02, 0.23)	0.48∗∗∗ (0.39, 0.60)	0.83+ (0.66, 1.03)	1.65∗∗∗ (1.32, 2.05)
Received food from government	1.21+ (0.99, 1.49)	0.19∗∗ (0.06, 0.32)	0.15∗ (0.01, 0.30)	0.14∗∗ (0.05, 0.23)	0.92 (0.76, 1.12)	1.34∗∗ (1.10, 1.63)	0.98 (0.82, 1.18)
Received cash from government	1.09 (0.93, 1.29)	0.06 (−0.04, 0.17)	0.14∗ (0.02, 0.26)	0.04 (−0.03, 0.12)	0.95 (0.80, 1.12)	0.89 (0.76, 1.04)	1.00 (0.86, 1.17)
Exposure to one or more interventions during lactation or for children							
Received only 1 intervention	1.70∗ (1.06, 2.73)	0.40∗∗ (0.15, 0.65)	0.38∗∗ (0.09, 0.66)	0.05 (−0.14, 0.24)	0.40∗∗∗ (0.27, 0.59)	0.77 (0.53, 1.13)	0.80 (0.54, 1.17)
Received ≥2 interventions	2.34∗∗∗ (1.48, 3.71)	0.66∗∗∗ (0.42, 0.90)	0.59∗∗∗ (0.32, 0.87)	0.22∗ (0.03, 0.40)	0.38∗∗∗ (0.26, 0.55)	0.99 (0.69, 1.43)	1.04 (0.71, 1.51)
Received all 3 interventions	3.54∗∗∗ (2.10, 5.97)	1.10∗∗∗ (0.81, 1.39)	1.07∗∗∗ (0.74, 1.40)	0.51∗∗∗ (0.29, 0.73)	0.24∗∗∗ (0.15, 0.38)	0.98 (0.63, 1.53)	1.51+ (0.96, 2.37)

Abbreviations: CI, confidence interval; IYCF, infant and young child feeding; MDD, minimum dietary diversity; NCD, noncommunicable disease; OR, odds ratio.

+*P* < 0.10; ∗*P* < 0.05; ∗∗*P* < 0.01; ∗∗∗*P* < 0.001.

1 All models were controlled for covariates including wealth, caste, women education, women employment status, women age, child age, and state fixed effects. 95% CI in parenthesis. *N* = 2847 children 12–24 mo.

2 Minimum dietary diversity for children is defined as consuming ≥5 of 8 food groups (WHO and UNICEF, 2021).

3 NCD-risk score modified for young children (range: 0–7) is the mean score of consumption of 6 food unhealthy groups for young children, which differs from the adult score in the following ways: it does not include unprocessed red meat, adds 2 additional types of sweetened beverages not recommended for IYC, and groups unhealthy sweet foods and unhealthy salty snacks together. These modifications to the score for IYC were made to better align with the IYCF unhealthy food and sweet beverage indicators published by WHO and UNICEF, 2021.

Exposure to multiple programs indicated a stronger association with outcomes among children. For example, compared with children who were not exposed to any programs, those exposed to all 3 programs were 3.5 times more likely to have achieved MDD, followed by 2.3 times among those exposed to 2 programs, and 1.7 times among those exposed to only 1 program. Similar dose–response relationships were observed between combined exposure and DDS, NCD-protect score, and zero vegetable or fruit consumption (Table 4).

## Discussion

In the realm of nutrition-sensitive program surveillance, the ability to evaluate diet quality is crucial. Typically, barriers to diet data collection, especially within programmatic monitoring and evaluation, have been prohibitive owing to high costs, expertise, and training requirements to gather dietary data [36]. Our study tackled the existing challenges in data collection for dietary assessments using a swift 5-min tool (DQQ) that efficiently gathered data on indicators of diet quality, covering both healthy and unhealthy consumption, for both mothers and their children. Our study revealed several key insights into the dietary patterns and the influence of SBCC and NSSP programs on maternal and child diet quality.

### Diet patterns among mothers and children

Consistent with previous research [17,37], our study found that maternal and child diets in India are characterized by a lack of dietary diversity and inadequate consumption of key food groups, including animal-source foods, fruits, and vegetables. Alarmingly, merely 12% of mothers consumed All-6 food groups recommended for Indians. In a population with low dietary diversity because it relates to both nutrient adequacy and health protection, additionally worrisome is the evidence of high consumption of unhealthy foods such as sweet, salty and fried snacks, and ultraprocessed foods. These findings underscore the urgent need for targeted interventions aimed at promoting consumption of healthy foods while concurrently discouraging the intake of unhealthy options. Addressing these issues requires multifaceted approaches by leveraging existing platforms for health promotion and social protection, ultimately contribute to tackling the double burden of malnutrition in the country with persistent undernutrition and doubling of adult overweight/obesity in the last decade [38].

In our study, children exhibited lower consumption across majority of food groups than mothers, and a much lower percentage of children had MDD than mothers, suggesting that food that is available in the household may not be fed to children for reasons that have to do with care practices. Previous studies reported great influence of parental dietary habits on dietary behaviors of their children regardless of demographic characteristics such as gender, age, socioeconomic status, and country, mainly owing to the role modeling and moderate restriction [39,40]. Additional research is needed to understand potential cultural barriers and broader constraints that may prevent mothers from feeding their children certain nutritious foods.

### Association between SBCC counseling and diet quality

In our study, exposure to counseling on health and nutrition demonstrated a positive association with higher consumption of healthy foods, with more significant associations with larger magnitudes in children than those in women. SBCC was associated with double the odds of children consuming MDD and half the odds of zero vegetables or fruits. These findings suggest that SBCC could help in improving child feeding practices, which is in congruence with the existing literature [41]. The potential to benefit for women may be less pronounced, as lack of cash or food availability may pose more binding constraints for them to be able to consume healthier diets. In fact, our findings showed that the simultaneous provision of SBCC alongside cash transfer and food supplements exhibited a markedly stronger association with both maternal and child diet quality outcomes. The additive effects observed underscore the importance of comprehensive interventions that address multiple facets determinants of diet.

The beneficial effects of counseling on enhancing dietary diversity for women aligned with findings from studies in several LMICs such as India, Malawi, Nepal, Bangladesh, Tanzania, and Ethiopia where MDD for women was ∼2 times higher among pregnant women or mothers exposed to nutrition counseling [[42], [43], [44], [45], [46], [47]]. Similarly, for children, the positive association of nutritional counseling on child dietary diversity are reported in both cross-sectional studies (by 0.6 food groups in Pakistan or 3 times higher in MDD in Ethiopia) [48,49] and randomized controlled trials in Bangladesh, Burkina Faso, or Malawi (effect size ranged from 2 to 6 percentage points) [[50], [51], [52]]. Understanding the differences in pathways between SBCC and improved diet practices for mothers and children could help develop programs for improving diet quality for all.

There is, however, limited evidence on the link between counseling and unhealthy food consumption. Typically, most counseling in LMICs focuses on improving frequency and variety of healthy food consumption but has not yet addressed unhealthy diet habits. This gap could potentially explain the lack of association found in our study: SBCC was not associated with lower consumption of unhealthy foods. Interestingly, in our study, SBCC was associated with higher odds of sweet beverage consumption among children. In this population, a vast majority of sweet beverages were “sweetened tea, coffee, or milk drinks,” of which the most commonly consumed beverage culturally is *chai* (tea). Greater feeding of sweet beverages (likely *chai*) also explains the small significant positive association between sweet beverage consumption and NCD-risk factor for children. Although tea contains milk, it also typically contains sugar that is not recommended for children, as it displaces crucial calories that need to be reserved for nutrient-rich food consumption.

In the context of nutrition transition, it is important to include counseling on unhealthy diet, particularly pertaining to ultraprocessed foods; in this population, special attention may be needed to counteract feeding sweet beverages. Findings from African and Latin American countries indicate snack foods and sugar-sweetened beverages contribute to 13%–38% of the total energy intake of children younger than 2 y [53]. There is a dearth of such evidence in the South Asian countries. To improve maternal and child diet, enhancing both counseling coverage and quality, with prioritization for those in need, is crucial. Many programs have targeted implementation and monitoring of nutrition interventions to infants and young children, or to women during pregnancy or postpartum, but not both [54]. There is a need to tailor culturally resonant counseling on diet during pregnancy and lactation, targeting both mothers and young children. For mothers, however, our results suggest that social protection programs beyond SBCC are needed to remove income-related and access-related barriers to healthy diets.

### Association between NSSP programs and diet quality

Although previous research has extensively linked cash or food transfers with DDS or MDD [11,12], limited literature has explored the association of NSSP programs with risk factor for NCDs or not using indicators such as NCD-protect and NCD-risk. In our study, receipt of cash and food were both found to be associated with higher consumption of healthy food, and cash was also associated with unhealthy food consumption among mothers, although at small magnitude. The associations of cash and food receipt with children’s diets was smaller in magnitude, but it was notable that receipt of food was associated with 34% greater odds of unhealthy food consumption among children.

The positive findings in our study are similar to those found in other intervention studies in LMICs between 2010 and 2020, wherein including cash, in-kind, and voucher programs targeting women and children improved dietary diversity and enhanced intake of micronutrient-rich foods. Various nutrition markers were positively impacted, with a more pronounced effect was observed among women than that among children [55]. Moreover, a study in Myanmar demonstrated sustained effects of cash transfers on maternal and child diets over the long term. Even during the COVID-19 pandemic, households that received transfers before the pandemic exhibited increased dietary diversity among women and children compared with control groups [56].

Our findings also suggest a small but positive link between cash transfers and mothers’ NCD-risk scores, aligning with results from Mexico, where cash or food transfers notably increased women’s BMI (in kg/m^2^), among existing overweight population [57]. These findings suggest the need to consider programmatic impact on both healthy and unhealthy foods; in particular, counseling may need a greater focus around unhealthy foods. They also point to the influence of the food environment on food choice, and the likely ubiquity of unhealthy foods, given that cash transfers were associated with higher unhealthy food consumption.

An important highlight of our study is the additive effect of combination of NSSP and health and nutrition programs on diet quality, where more interventions exposure is associated with higher diet quality. Literature indicates greater benefits of a combination of modalities of SBCC interventions than those of only 1 type of SBCC modality [58]. Evidence from a randomization trial in Bangladesh also suggested that cash transfers and nutrition SBCC improved nutritional status (a nearly 8-percentage point decrease in stunting prevalence), with the key underlying mechanisms being attributed to improved diets [59]. Our findings underscore the importance of comprehensive approaches that combine education, resource provision, and targeted support to maximize impact in promoting healthy dietary practices among both children and women.

### Strength and limitations

Our study has several strengths, including the ability to holistically assess diet quality for both mothers and children in the context of COVID-19 pandemic. Given logistical challenges posed by the pandemic, conducting face-to-face interviews or physical assessments were not feasible or safe. The DQQ questionnaire proved to be culturally relevant and feasible, offering a concise and easily understandable format that minimized respondent burden and maximized participation rates. Having dietary data for both mothers and children provides crucial information to support the focus of many maternal and child health and nutrition programs during the entire span of the first 1000 d.

However, we also acknowledge some limitations. First, phone surveys may introduce bias, particularly among marginalized populations with limited access to mobile phones. Our study sample had a higher education level than the sample from the national representative survey, indicating selection bias. Second, although we used random sampling from phone number lists and reported refusal rates, we were unable to account for population size in selecting the rural districts and villages, which might have affected the representativeness of our findings. Third, despite conducting cognitive interviews and revising the questionnaire in measuring NSSP participation [34], concerns persist regarding the accuracy of women’s recall of transfers received during their pregnancy. For counseling during pregnancy and IYCF, we simply asked mothers whether a health care provider discussed health and nutrition topics with them, without inquiring about specific messages received to alleviate the burden on respondents in a phone survey. Finally, the study did not include the full IYCF DQQ because it was not yet available at the time of the survey. Indicators related to the construct of “healthy diets that prevent NCDs” include NCD-protect and NCD-risk, which align with the WHO global dietary guidance for the general population, but the meaning of these indicators is not well established for IYC. In this study, we included them for both mothers and children primarily to understand how diets correspond or differ within mother–child dyads, where NCD-protect functions as a count of healthy and diverse plant foods and NCD-risk functions as a count of unhealthy foods. We therefore modified the NCD-risk indicator for children to correspond more closely with the WHO and UNICEF IYCF indicators on unhealthy practices and include other IYCF-specific indicators: “zero vegetables or fruits,” “sweet beverage consumption,” and “unhealthy food consumption,” which measures the intake of sweet, fried, salty, or ultraprocessed snack foods.

In conclusion, maternal and child diet quality was suboptimal, but maternal diets were more likely to have adequate diversity. SBCC had greater positive impact on IYCF than food and cash transfers, while cash generally had a stronger association with improved maternal diets. Food and cash also associated with higher risk of unhealthy food consumption. As the number of interventions received increased, dietary diversity and consumption of recommended food groups improved, but unhealthy food consumption was not affected. These results suggest 3 main actions: first, food and cash transfers (especially cash) appear to be important in reducing real income barriers to healthy diets in mothers; second, knowledge and practices are a significant barrier to adequate child diets—where food resources exist in the household, they are not always fed to young children, and our study reveals significant positive associations between SBCC and improved child diets; and third, SBCC should increase attention to counseling on avoiding unhealthy food consumption, particularly unhealthy foods and sweet beverages for children. Policy responses should leverage both educational strategies, such as SBCC, and address practical barriers through increased resources, such as cash and food availability, to improve diet quality for mothers and children.

## Author contributions

The authors’ responsibilities were as follows – PHN, AH: conceptualized the article and wrote significant parts of the article; SN, AP: supported data analyses and drafted parts of the article; RA: supervised data collection and drafted parts of the article; PHN, RA, RA: critically reviewed the article; and all authors: read and approved the final manuscript.

## Conflict of interest

PHN is a member of the Journal’s editorial board. The authors report no conflicts of interest.

## Funding

Funding for this study was provided by the Bill & Melinda Gates Foundation, through DataDENT, managed by Johns Hopkins University. Additional financial support to the study was provided by the Transforming Agri Food System in South Asia (TAFSSA)—a CGIAR initiative led by the International Food Policy Research Institute, and the Swiss Agency for Development and Cooperation. The funders had no role in study design, data collection and analysis, data interpretation, decision to publish, or preparation of the manuscript.
