# Supplementary material for: Diet Quality Among Mothers and Children in India: Roles of Social and Behavior Change Communication and Nutrition-Sensitive Social Protection Programs
Source: J Nutr. 2024 Jul 23;154(9):2784–94. doi: 10.1016/j.tjnut.2024.07.026 (PMC11393167; doi:10.1016/j.tjnut.2024.07.026)
Supplement: Multimedia component 1 [file mmc1.docx]

**Supplemental Table 1: Characteristics of the study sample compared to those of the sample in the 2019-2021 National Family Health Survey**

|  | **Chhattisgarh** | | **Gujarat** | | | **Madhya Pradesh** | | | **Odisha** | | | **Telangana** | | | **Uttar Pradesh** | | |  |
| --- | --- | --- | --- | --- | --- | --- | --- | --- | --- | --- | --- | --- | --- | --- | --- | --- | --- | --- |
|  | Current study | NFHS-5 | | Current study | NFHS-5 | | Current study | NFHS-5 | | Current study | NFHS-5 | | Current study | NFHS-5 | | Current study | NFHS-5 | |
| Sample size | 1114 | 28,468 | | 1048 | 33,343 | | 1454 | 48,410 | | 988 | 27,971 | | 1027 | 27,518 | | 996 | 93,124 | |
| Mother’s age (years) |  |  | |  |  | |  |  | |  |  | |  |  | |  |  | |
| 15-19 | 0.2 | 16.9 | | 1.0 | 15.6 | | 1.8 | 17.9 | | 1.7 | 14.1 | | 1.6 | 12.3 | | 0.6 | 21.0 | |
| 20-24 | 44.9 | 16.5 | | 49.3 | 16.1 | | 50.8 | 17.4 | | 32.6 | 16.3 | | 44.8 | 14.9 | | 35.4 | 18.5 | |
| >24 | 55.1 | 66.7 | | 50.1 | 68.2 | | 46.5 | 64.6 | | 65.6 | 69.6 | | 53.9 | 72.7 | | 63.7 | 60.6 | |
| Mother’s education (number of years of schooling completed) |  |  | |  |  | |  |  | |  |  | |  |  | |  |  | |
| No schooling | 2.2 | 22.2 | | 12.7 | 20.9 | | 10.6 | 25.5 | | 2.7 | 21.9 | | 1.9 | 32.6 | | 11.9 | 28.6 | |
| <5y | 1.8 | 4.4 | | 8.8 | 7.1 | | 6.1 | 4.7 | | 3.3 | 8.1 | | 1.5 | 3.2 | | 2.9 | 2.3 | |
| 5-9y | 40.6 | 36.5 | | 49.7 | 38.2 | | 51.1 | 40.5 | | 34.9 | 37.1 | | 11.7 | 18.7 | | 39.8 | 29.8 | |
| 10-11y | 22.7 | 13.5 | | 12.8 | 12.4 | | 15.3 | 11.0 | | 31.9 | 15.8 | | 32.8 | 19.0 | | 13.2 | 11.7 | |
| 12y or more | 32.3 | 23.4 | | 15 | 21.3 | | 15.6 | 18.3 | | 25.9 | 17.2 | | 50.1 | 26.5 | | 30.6 | 27.6 | |
| Caste |  |  | |  |  | |  |  | |  |  | |  |  | |  |  | |
| Scheduled caste or tribe | 38.4 | 47.2 | | 23.4 | 29.1 | | 47.5 | 38.7 | | 26.2 | 44.2 | | 35.4 | 30.7 | | 37.6 | 27.7 | |
| Other backward classes | 59.5 | 44.6 | | 61.4 | 44.2 | | 42.2 | 42.8 | | 49.9 | 34.5 | | 53.5 | 57.3 | | 45.3 | 50.4 | |
| Other caste categories | 1.0 | 8.2 | | 14.4 | 25.7 | | 10.3 | 17.5 | | 23.6 | 20.9 | | 10.5 | 11.2 | | 17.0 | 21.7 | |
| Household Assets |  |  | |  |  | |  |  | |  |  | |  |  | |  |  | |
| Television | 90.8 | 69.5 | | 69.1 | 74.3 | | 71.2 | 60.5 | | 86.8 | 59.0 | | 90.8 | 84.0 | | 45.1 | 53.9 | |
| Mobile telephone | 95.8 | 85.7 | | 95.7 | 92.4 | | 95.1 | 89.6 | | 96.3 | 88.3 | | 97 | 92.5 | | 93.4 | 94.9 | |
| Computer | 7.1 | 6.7 | | 2.9 | 8.3 | | 1.7 | 5.6 | | 3.7 | 4.8 | | 8.1 | 6.0 | | 1.9 | 6.3 | |
| Refrigerator | 21.9 | 22.5 | | 50.3 | 52.6 | | 18.8 | 23.7 | | 42.9 | 24.0 | | 49.9 | 41.1 | | 21.3 | 29.3 | |

Note: NFHS – National Family Health Survey

**Supplemental Table 2: Summary statistics of diet quality indicators among mothers and children**

| **Indicators** | **Mother**  **(n=6627)** | **Children 6-24 months**  **(n=4865)** |
| --- | --- | --- |
| **Dietary Diversity Score (DDS), (mean ±SD)** | **5.0±1.9** | **3.8 ± 1.7** |
| **Minimum dietary diversity – Women (MDD-W)^1^, %** | **57.0** | **33.0** |
| Breastmilk | -- | 91.7 |
| Starchy food | 99.4 | 83.1 |
| Legumes | 72.1 | -- |
| Nuts and seeds | 33.4 | -- |
| Legumes and nuts | -- | 48.3 |
| Dairy | 54.4 | 57.6 |
| Flesh food | 17 | 5.6 |
| Eggs | 26.3 | 12.3 |
| Dark green leafy vegetables | 54.1 | -- |
| Vitamin A-rich fruits and vegetables | 26 | 29.7 |
| Other veggies | 72.2 | -- |
| Other fruits | 41.6 | -- |
| Other fruits and veggies | **--** | 52.1 |
| **NCD -Protect score^2^, (0-9) (mean ±SD)** | **3.9±1.7** | **2.3±1.8** |
| Whole grains | 78.9 | 58.4 |
| Legumes | 72.1 | 46 |
| Nuts and seeds | 33.4 | 11.2 |
| Vitamin A-rich orange vegetables | 15.7 | 9.2 |
| Dark green leafy vegetables | 54.1 | 19.8 |
| Other vegetables | 72.2 | 31.7 |
| Vitamin A-rich fruits | 18 | 11 |
| Citrus | 7.4 | 4.1 |
| Other fruits | 39.4 | 34.6 |
| **NCD – Risk score^3^ (0-9) (mean ±SD)** | **1.2±1.3** | **1.0±1.0** |
| Soft drinks (sodas) | 2 | 1.2 |
| Fruit juice and fruit drinks | -- | 7.7 |
| Sweetened tea/coffee/milk drinks | -- | 37.7 |
| Grain-based sweets | 47.1 | -- |
| Other sweets | 11.8 | -- |
| Sweet foods | -- | 31 |
| Processed meat | 0.8 | 0.4 |
| Unprocessed red meat | 4.3 | -- |
| Deep fried food | 18.4 | 19.8 |
| Fast food & Instant noodles | 5.2 | -- |
| Packaged ultra-processed salty snacks | 27.3 | -- |
| **All-5 globally recommended food groups^4^, %** | **29.8** | **10.3** |
| At least one vegetable | 84.7 | 41.5 |
| At least one fruit | 49 | 39.5 |
| At least one pulse, nut, or seed | 79.1 | 48.3 |
| At least one animal-source food | 67.7 | 45.7 |
| At least one starchy staple | 99.4 | 83.1 |
| All five food groups | 29.8 | 10.3 |
| **All-6 food groups recommended in India^5^, %** | **12.2** | 3 |
| At least one cereal | 99.2 | 80.6 |
| At least pulse/eggs/fish/meat | 83.1 | 52 |
| At least milk or curd | 53.7 | 36.9 |
| At least one vegetable | 84.7 | 41.5 |
| At least one fruit | 49 | 39.5 |
| At least one nut and seed | 33.4 | 11.2 |

IYCF: Infant and young child feeding, MDD-W: Minimum dietary diversity for women. NCD: Non-communicable disease.

^1^ Minimum dietary diversity for women is defined as consuming at least 5 out of 10 food groups (FAO 2021).

^2^ NCD-Risk score is defined as mean score of consumption of 8 food groups to limit that may increase mother’s vulnerability to NCDs, with a range of 0-9 (processed meat is double weighted). NCD-Risk score modified for young children (range 0-7) is the mean score of consumption of 6 food unhealthy groups for young children, which differs from the adult score in the following ways: it does not include unprocessed red meat, adds two additional types of sweetened beverages not recommended for IYC, and groups unhealthy sweet foods together and unhealthy salty snacks together. These modifications to the score for IYC were made to better align with the IYCF unhealthy food and sweet beverage indicators published by WHO and UNICEF 2021.

^3^ The five food groups are starchy staples; vegetables; fruits; pulses, nuts or seeds; animal-source foods.

^4^ The six food groups are cereal grains; vegetables; fruits; nuts or seeds; pulses, eggs, fish, or meat; dairy.

**Supplemental Questionnaire**

**Phone survey to study the impact of COVID-19 on food security and utilization of nutrition services among mothers of children under two years in India**

*[Note for the interviewer: The following is to determine if the phone is on speaker]*

**Read out:** I’m having a bit of trouble with the phone connection. Is the phone on speaker on your side? It’s not a problem if it is, but I just need to adjust a setting if it is.

Answer:

1. No
2. Yes

**MODULE A: ADMINISTRATIVE**

| **Q. No.** | **Administrative** | **Response** |
| --- | --- | --- |
|  | Respondent’s ID |  |
|  | State Name / Code | 1. Chhattisgarh 2. Gujarat 3. Madhya Pradesh 4. Odisha 5. Telangana 6. Uttar Pradesh |
|  | District Name / Code |  |
|  | Block Name / Code |  |
|  | Village Name / Code |  |
|  | Survey Date |  |
|  | Time Started/ Ended |  |
|  | Result of the interview | 0. Did not agree to give interview  1. Interview completed  2. Wrong phone number  3. Phone number is not reachable/switched  off/not picking up  4. Requested to reschedule but not picking up/ not  reachable/switched off  5. Respondent dead  6. Child dead  7. Contact number not available  21. Half-completed: Respondent refused to  continue with survey  22. Half-completed: Call dropped mid-session  (tried 3 separate time but not picked-up) |

**MODULE B: PERSONAL AND HOUSEHOLD SCHEDULE**

| **Q. No.** | **Questions** | **Response** |
| --- | --- | --- |
|  | Mothers Name / What is your name? |  |
|  | What is your age? | 1. ___ ___  (Record age in completed years 18-45)  -99. Don’t know |
|  | What is the highest grade or education level you completed? | -88. Never attended school  0. Started school, but not completed class 1  1. Completed class 1  2. Completed class 2  3. Completed class 3  4. Completed class 4  5. Completed class 5  6. Completed class 6  7. Completed class 7  8. Completed class 8  9. Completed class 9  10.Completed class 10  11. Completed class11  12. Completed class 12  13. Bachelors  14. Master/PhD  15. Non-formal education  16. Technical/ vocational-99. Do not know |
|  | Do you belong to a schedule caste, a schedule tribe, other backward class, or none of these? | 1. Schedule caste 2. Schedule tribe 3. Other backward caste (OBC) 4. General category 5. Don’t know |
|  | What is your main occupation? | 1. Professional (technical, administrative, and managerial) 2. Clerical 3. Sales worker 4. Agriculture worker 5. Service worker 6. Production worker 7. Home maker/housewife   95. Other (specify)  -99. Don’t know |
|  | Date of birth of the youngest child | ___ ____ ___ ___ ___ ___ ___ ___  DD MM YYYY  -99. Don’t know |
| 206a | Name of the youngest child | __________________________ |
|  | How many members live in this household? | 1. ___ ____  (Number of members 2-20)  -99. Don’t know |

**MODULE C:** **EXPOSURE TO SERVICES**

**3.1. Household-level social protection**

| **Q. No.** | **Questions** | **Responses** |
| --- | --- | --- |
|  | Have you heard of a ration shop (society/quota/control) from where you can buy rice, wheat, kerosene at low cost? | 1. Yes   0. No  -99. Don’t know |
|  | Does your household have a Ration card? | 1. Yes  0. No🡪 308  -99. Don’t know |
|  | Did you or anyone in your household ever purchase rice, wheat, sugar, or kerosene from a ration shop (society/quota/control) with your ration card? | 1. Yes  0. No 🡪 308  -99. Don’t know🡪 308 |
| 304. | For how long has your household been purchase items from the ration shop? | 1. More than 2 years 🡪306  2. 1-2 years-🡪 306  3. <1 year |
| 305. | **Ask if Q304 = 3**  If less than 1 year, how many months ago did someone in your household buy items from the ration shop? | __ __ month (0-11)    -66. A few months ago  -99. Don’t know |
| 306. | **Ask if Q304 = 1, 2 or 3**  What does your household usually purchase from the ration shop (society/quota/control) using ration card? Multiple code | 1. Rice  2. Wheat  3. Sugar  4. Kerosene  5. Oil  6. Millets  7. Salt  8. Lentils  -95. Other(specify)____________ |
| 307. | When was the last time someone in your household bought items from the ration shop (society/quota/control)? | 1.__ __ month (0-11)  __ __ year (0-5)  -66. A few months ago  -99. Don’t know |
| 308. | **Ask if Q302 = 1, 0 or -99; if Q303 = 0 or -99**  In the last one year, did anyone in your household receive food grains from the government for any reason? | 1. Yes  0. No 🡪 311  -99. Don’t know🡪311 |
| 309. | What food grains did your household receive? | 1. Wheat  2. Rice  3. Pulse  95.Other (specify)____________ |
| 310. | When was the last time your household received food grains from government? | __ __ month (0-11)  __ __ year (0-5)  -66. A few months ago  -99. Don’t know |
| 311. | **Ask if Q308 = 1, 0 or -99**  Did your household receive food grains from the government because of the coronavirus crisis? | 1. Yes  0. No 🡪 313  -99. Don’t know 🡪 313 |
| 312. | Where did you get the food grains from? | 1. Ration shop  2. Panchayat office  3. From Anganwadi worker  4. Home distribution  5. Community Camp / event  95.Other (specify)_____________ |
| 313. | **Ask if Q311 = 1, 0 or -99**  In the last one year, did anyone in your household receive cash from government for any reason? | 1. Yes  0. No 🡪315  -99. Don’t know 🡪315 |
| 314. | When was the last time your household received cash from government? | __ __ month (0-11)  __ __ year (0-5)  -66. A few months ago  -99. Don’t know |
| 315. | **Ask if Q313 = 1, 0 or -99**  Did your household receive cash from the government because of the coronavirus crisis? | 1. Yes  0. No  -99. Don’t know |
| 316. | Does anyone in your HH work under MGNREGA? | 1. Yes  2. Not heard about MGNREGA  3. No, do not have a job card  0. No despite having a job card  -99. Don’t know |

**3.2** **Services during last pregnancy**

| **Q. No.** | **Questions**  ***While you were pregnant with [NAME of the youngest child]*** | **Responses** |
| --- | --- | --- |
| 317. | How many times did you attend VHND or other community group-events ? | ___ ___  (No. of times attended 0-15)  -99. Don’t know |
| 318. | How many times did AWW/ASHA/FLWs visit your home? | ___ ___  (No. of times attended 0-15)  -99. Don’t know |
| 319. | How many times did you receive ANC? | ___ ___  (No. of times attended 0-20)  -99. Don’t know |
| 320. | **Ask only if 319 ≥1**  Were you weighed during any of these ANC visits? | 0. No  1. Yes  -99. Don’t know |
| 321. | How many IFA tablets did you receive? | 1. ___ ___ ___  (No. of tablets 0-300)  -99. Don’t know |
| 322. | Did you receive deworming tablet? | 0. No  1. Yes  -99. Don’t know |
| 323. | Did you receive tetanus vaccination? | 0. No  1. Yes  -99. Don’t know |
| 324. | During your pregnancy with *[NAME*], did a health care provider or community health worker (such as ASHA/AWW/ANM) talk with you about your health and nutrition (such as weight, iron tablet or syrup, or foods to eat during pregnancy)? | 0. No  1. Yes  -99. Don’t know |
| **Pregnancy Social Protection Questions when you were pregnant with *[Name of the youngest child]*** | | |
| 325. | Did you receive any food from the government or AWC for you during your last pregnancy? | 0. No🡪 327  1. Yes  -99. Don’t know🡪 327 |
| 326. | What did you receive from the government / AWC during your last pregnancy?  Multiple choice | 1. Dry ration (rice/dal)  2. Cooked food  3. THR (e.g., Daliya, khichdi mix)  95. Other (specify)_ |
| 327. | During your last pregnancy, did you receive cash from government because you were pregnant? | 0. No  1. Yes  -99. Don’t know |
| 328. | Did you receive cash from government because you delivered at the health centre? | 1. No, despite delivering at a health center 2. No, did not deliver at health center   2. Yes  -99. Don’t know |

**3.3.** **Services for her last child (0-24 months)**

***Note to enumerator: For child related services, please recall for [NAME OF THE YOUNGEST CHILD].***

| **Q. No.** | **Questions**  ***Please ask for the service for last month and April and May 2021 for each question below*** | **Responses** | |
| --- | --- | --- | --- |
|  |  | **A. In last month (30 days?)**  0. No  1. Yes  -99. Don’t know | **B. In April-May 2021? ASK only for children >6months**  0. No  1. Yes  -99. Don’t know |
| 329. | Did you attend any Village Health and Nutrition Day (VHND) or other community event? |  |  |
| 330. | Did AWW/ASHA visit your home to check on your child? |  |  |
| 331. | Was your child’s height or weight measured by AWW/ASHA? |  |  |
| 332. | Did your child receive immunization services? |  |  |
| 333. | Did your child or you receive IFA supplements?  **ASK only for children 6 months** |  |  |
| 334. | Did your child receive vitamin A supplements?  **ASK only for children >9 months** |  |  |
| 335. | Did your child receive deworming tablets/syrup?  **ASK only for children >12months** |  |  |
| 336. | Did any health care provider or community worker (such as ASHA/AWW/ANM) talk with you about feeding (breastfeeding or complementary feeding) your child? |  |  |

|  | **Mother/Child Food or cash programs** | |
| --- | --- | --- |
| 337. | Did you receive any food from the government/AWC for you after the ***[NAME of the youngest child***] was born? | 0. No  1. Yes  -99. Don’t know |
| 338. | Did you receive cash from government for ***[NAME of the youngest child***]? | 0. No  1. Yes  -99. Don’t know |
| 339. | Have you received cash from the government because [***[NAME of the youngest child*** was vaccinated? | 0. No  1. Yes  -99. Don’t know |
| 340. | **If Q 338=1 OR Q 339=1 then ask**  When was the last time you received cash from the government for you or your child ***[NAME of the youngest child***] | 1__ month (0-11)  __ year (1-2)  -66. A few months ago  -99. Don’t know |
| 341. | **Ask ONLY for children older than 6 months**  Did you receive any food from the government/AWC for your child? | 0. No🡪 401  1. Yes  -99. Don’t know🡪 401 |
| 342. | When was the last time you received food from the government/AWC for ***[NAME of the youngest child***]? | __ month (0-11)  __ year (1-2)  -66. A few months ago  -99. Don’t know |

**MODULE D: FOOD INSECURITY**

*Note to enumerator*: *For each of the following questions, consider what has happened in the past 30 days*

**Read Out:** Now I will ask you questions about your and your household members’ access to food in the last 30 day.

| **Q. No.** | **Questions: During the last one month, was there a time when, because of lack of money or other resources:** | **Responses**  0. No  1. Yes  99. Don’t know |
| --- | --- | --- |
|  | You or any member of household, were worried you would not have enough food to eat? |  |
|  | You or any member of household, were unable to eat healthy and nutritious food? |  |
|  | You or any member of household, ate only a few kinds of foods? |  |
|  | You or any member of household, had to skip a meal? |  |
|  | You or any member of household, ate less than you thought you should? |  |
|  | Your household ran out of food? |  |
|  | You or any member of household, were hungry but did not eat? |  |
|  | You or any member of household, went without eating for a whole day? |  |

**MODULE E:** **WOMEN’S DIETARY INTAKE**

Interviewer reads: Now I’d like to ask you some yes-or-no questions about foods and drinks that you consumed yesterday during the day or night, whether you had it at home or somewhere else.

First, I would like you to think about yesterday, from the time you woke up through the night. Think to yourself about the first thing you ate or drank after you woke up in the morning … Think about where you were when you had any food or drink in the middle of the day … Think about where you were when you had any evening meal … and any food or drink you may have had in the evening or late-night... and any other snacks or drinks you may have had between meals throughout the day or night.

I am interested in whether you had the food items I will mention even if they were combined with other foods. Please listen to the list of foods and drinks, and if you ate or drank ANY ONE OF THEM, say yes.

| **Q. No.** |  | **Food items** | 0. No  1. Yes |
| --- | --- | --- | --- |
|  | *(Do not read food*  *group names)* | **Yesterday, did you eat any of the following foods?** |  |
|  | *01 staple foods made*  *from grains* | Rice, idli, dosa, poha, naan, kulcha, paratha, or upma? |  |
|  | *02.1 whole grains* | Chapati, roti, dalia, or roasted maize? |  |
|  | *02.2 whole grains* | Pearl millet or finger millet? |  |
|  | *03 white roots/tubers* | Potato, sweet potato, turnip, arum root, tapioca, or raw banana? |  |
|  | *04 legumes* | Daal, sambar, chickpeas, kidney beans, soya, or khichdi? |  |
|  |  | **Yesterday, did you eat any of the following vegetables?** |  |
|  | *05 vitamin A-rich*  *orange veg* | Carrots or pumpkin that is orange inside? |  |
|  | *06.1 dark green leafy*  *Vegetables* | Mustard leaves, spinach, radish leaves, cassava leaves, taro leaves, drumstick leaves, amaranth leaves, or wild greens/other greens? |  |
|  | *07.1 other vegetables* | Tomatoes, eggplant, okra/lady finger, French beans, cauliflower, cabbage, or beetroot? |  |
|  | *07.2 other vegetables* | Bitter gourd, bottle gourd, pointed gourd, ivy gourd, apple gourd, ridged gourd, or snake gourd? |  |
|  | *07.3 other vegetables* | Cucumber, radish, capsicum, German turnip, or drumstick? |  |
|  |  | **Yesterday, did you eat any of the following fruits?** |  |
|  | *08 vitamin A-rich fruits* | Papaya, mango, orange musk melon, or apricots? |  |
|  | *09 citrus* | Orange, tangerine, or grapefruit? |  |
|  | *10.1 other fruits* | Ripe banana, apple, pear, watermelon, guava, custard apple, pomegranate, or pineapple? |  |
|  | *10.2 other fruits* | Grapes, kiwi, peaches, jackfruit, chickoo, jamun, palmyra palm fruit, or other wild fruits? |  |
|  |  | **Yesterday, did you eat any of the following sweet?** |  |
|  | *11 grain sweets* | Cakes, cream biscuits, biscuits, suji halwa / kesari bath, jalebi, or ladoo? |  |
|  | *12 other sweets* | Other mithai, rice pudding, kulfi, ice cream, milkshake, toffees, or chocolates? |  |
|  |  | **Yesterday, did you eat any of the following foods of animal origin?** |  |
|  | *13 eggs* | Eggs? |  |
|  | *14 cheese* | Paneer or cheese? |  |
|  | *15 yogurt* | Curd, lassi, buttermilk, or raita? |  |
|  | *16 processed meat* | Sausages or salami? |  |
|  | *17 unprocessed red*  *meat (ruminant)* | Mutton, beef, lamb, or liver? |  |
|  | *18 unprocessed red*  *meat (non-ruminant)* | Pork or wild meat? |  |
|  | *19 poultry* | Chicken, duck, or turkey? |  |
|  | *20 fish & seafood* | Fish, prawn, crab, or seafood? |  |
|  |  | **Yesterday, did you eat any of the following other foods?** |  |
|  | *21 nuts & seeds* | Peanuts, cashews, almonds, pistachios, walnuts, pumpkin seeds, or sunflower seeds? |  |
|  | *22 ultra-processed*  *packaged salty snacks* | Potato chips, namkeen or mixture? |  |
|  | *23 instant noodles* | Instant noodles such as Maggi noodles or Wai Wai? |  |
|  | *24 deep fried foods* | Samosa, pakora, puri, vada, mathri, kachori, murukku, bonda? |  |
|  |  | **Yesterday, did you have any of the following beverages?** |  |
|  | *25 fluid milk* | Milk, flavoured milk, chai with milk, or coffee with milk? |  |
|  | *26 sweetened tea/*  *coffee/ milk drinks* | Tea with sugar, coffee with sugar, milk with sugar, flavoured milk, Bournvita, Horlicks, or Boost? |  |
|  | *27 fruit juice* | Fruit juice, packet juice such as Rasna or Frooti, sugarcane juice, or nannari sarbath? |  |
|  | *28 SSBs (sodas)* | Soft drinks such as Sprite, Pepsi, or Mirinda, or energy drinks? |  |
|  |  | Yesterday, did you get food from any place like |  |
|  | *29 fast food* | McDonald's, KFC, Pizza Hut, ec. |  |

**MODULE F:** **INFANT AND YOUNG CHILD FEEDING PRACTICES (FOR CHILDREN 0-24 MONTHS)**

| **No** | **Question** | **Response** |
| --- | --- | --- |
|  | Was [***[NAME of the youngest child***] ever breastfed? | 0. No 🡪 603  1. Yes |
|  | **Ask if Q601 = 1**  How long after birth was [***[NAME of the youngest child***] first put to the breast?  *If immediately, circle “000”*  *If less than one hour, record “00” hours*  *If less than 24 hours, record hours*  *Otherwise, record days* | *If immediately, circle “000”*  *If less than one hour, record “00” hours*  *If less than 24 hours, record hours*  *Otherwise, record days* |
|  | **Ask if Q601 = 0 or 1**  In the first 2 days after delivery, was [NAME] given anything other than breastmilk to eat or drink – anything at all like water, infant formula or baby milk, honey, or sugar water? | 0. No  1. Yes |
|  | Was ***[NAME of the youngest child***] breastfed yesterday during the day or at night? | 0. No 🡪 606  1. Yes |
|  | Did ***[NAME of the youngest child***] drink anything from a bottle with a nipple yesterday during the day or at night? | 0. No  1. Yes |

| Now I would like to ask you about liquids that [NAME] may have had yesterday during the day or at night. Please tell me about all drinks, whether [NAME] had them at home, or somewhere else.  Yesterday during the day or at night, did [NAME] have... | | | | | |
| --- | --- | --- | --- | --- | --- |
| ***(Do not read food group names)*** | |  | 0. No  1. Yes | |  |
|  | *Water* | 1. Plain water? | |  | |
|  | *Infant Formulas* | 1. Infant formula or baby milk, such as, Amul, Lactogen,   Dexolac? | |  | |
|  |  | IF YES: How many times did (NAME) drink infant formula? (IF 7 OR MORE TIMES, RECORD '7'). | | # | |
|  | *Milk* | 1. Milk from animals, including, fresh, tinned, or powdered? | |  | |
|  |  | IF YES: How many times did (NAME) drink milk? (IF 7 OR MORE TIMES, RECORD '7'). | | # | |
|  |  | IF YES: Was any of the milk a sweet or flavoured type of milk? | |  | |
|  | *Yogurt drinks* | 1. Lassi or buttermilk? | |  | |
|  |  | IF YES: How many times did (NAME) drink yogurt? (IF 7 OR MORE TIMES, RECORD '7'). | | # | |
|  |  | IF YES: Was the yogurt drink a sweet or flavored type of yogurt drink? | |  | |
|  | *Chocolate- flavored /*  *sweetened drinks* | 1. Milk with sugar, flavoured milk, Bournevita, Horlicks, or Boost? | |  | |
|  | *Fruit juice* | 1. Fruit juice, packet juice such as Rasna or Frooti, sugarcane juice, or nannari sarbath? | |  | |
|  | *Soft drinks* | 1. Soft drinks, such as, Sprite, Pepsi, Mirinda, or energy drinks? | |  | |
|  | *Tea, coffee, herbal*  *Drinks* | 1. Tea, coffee, or herbal drinks? | |  | |
|  |  | IF YES: Was the drink sweetened? | |  | |
|  | *Clear broth* | 1. Clear broth or clear soup? | |  | |
|  | *Any other liquids* | 1. Any other liquids? | |  | |
|  |  | IF YES: What was the drink? | |  | |
|  |  | IF YES: Was the drink sweetened? | |  | |
| Now I would like to ask you about foods that [NAME] had yesterday during the day or at night. I am interested in foods your child ate whether at home or somewhere else. Please think about snacks and small meals as well as main meals.  I will ask you about different types of foods, and I would like to know whether your child ate the food even if it was combined with other foods. Please do not answer ‘yes’ for any food or ingredient used in a small amount to add flavour to a dish.  Yesterday during the day or at night, did [NAME] eat: | | | | | |
|  | *Yogurt* | a. Curd or raita? | |  | |
|  | *Foods made from*  *Grains* | b1. Rice, idli, dosa, poha, naan, kulcha, paratha, or upma? | |  | |
|  |  | b2. Chapati, roti, dalia, or roasted maize? | |  | |
|  |  | b3. Pearl millet, finger millet, or ragi malt? | |  | |
|  | *Vitamin A-rich*  *orange vegetable* | c. Carrots or pumpkin that is orange inside? | |  | |
|  | *White roots/ tubers* | d. Potato, sweet potato, turnip, arum root, tapioca, or raw banana | |  | |
|  | *Dark Green Leafy*  *Vegetable* | e. Mustard leaves, spinach, radish leaves, cassava leaves, taro leaves, drumstick leaves, amaranth leaves, or wild greens/other greens | |  | |
|  | *Other vegetables* | f1. Tomatoes, eggplant, okra/lady finger, French beans, cauliflower, cabbage, or beetroot? | |  | |
|  |  | f2. Bitter gourd, bottle gourd, pointed gourd, ivy gourd, apple gourd, ridged gourd, or snake gourd? | |  | |
|  |  | f3. Cucumber, radish, capsicum, German turnip, or drumstick? | |  | |
|  | *Vit A-rich orange*  *Fruit* | g. Papaya, mango, orange musk melon, or apricots? | |  | |
|  | *Other fruits* | h1. Orange, tangerine, or grapefruit? | |  | |
|  |  | h2. Ripe banana, apple, pear, watermelon, guava, custard apple, pomegranate, or pineapple? | |  | |
|  |  | h3. Grapes, kiwi, peaches, jackfruit, chickoo, jamun, palmyra palm fruit, or other wild fruits? | |  | |
|  | *Organ Meats* | i. Liver or kidney? | |  | |
|  | *Processed meat* | j. Sausages or salami? | |  | |
|  | *Meat* | k. Any other meat, such as mutton, beef, lamb, pork, wild meat, chicken, or duck? | |  | |
|  | *Eggs* | l. Eggs? | |  | |
|  | *Fish and shellfish* | m. Fish, prawn, crab, or seafood? | |  | |
|  | *Legumes* | n. Daal, sambar, chickpeas, kidney beans, soya, or khichdi? | |  | |
|  | *Nuts and Seeds* | o. Peanuts, cashews, almonds, pistachios, walnuts, pumpkin seeds, or sunflower seeds? | |  | |
|  | *Cheese* | p. Paneer or cheese? | |  | |
|  | *Insects* | q. Termites, ants, or locusts? | |  | |
|  | *Sweets* | r. Any sweet foods such as cakes, biscuits, toffees, kulfi, rice pudding, jalebi, ladoo or other mithai? | |  | |
|  | *Savory and fried*  *Snacks* | S. Potato chips, samosa, pakora, puri, vada, mathri, kachori; or instant noodles such as Maggi noodles? | |  | |
|  | *Any other foods* | u. Any other solid, semi-solid, or soft food?  IF YES: What was the food?  MARK THE APPROPRIATE FOOD GROUP FOR EACH ADDITIONAL FOOD, IF THE GROUP IS NOT YET CODED ‘YES’.  IF UNABLE TO DETERMINE WHICH GROUP THE ADDITIONAL FOOD BELONGS TO, RECORD THE NAME OF THE FOOD. | |  | |
|  |  | How many times did [NAME] eat any solid, semi-solid or  soft foods yesterday during the day or night?  *If 7 or more times, record “7”*  *If number of times not known, record “9”* | | # | |

**MODULE G: INCOMES AND ASSETS**

**Read Out:** Next, I would like to ask you some information about recent changes in your income sources.

| **Q. No.** | **Question** | **Responses** |
| --- | --- | --- |
|  |  | 1. No effect 2. Lost existing job or business 3. Kept existing job or business, but decreased earning   -77. Refuse to answer |
|  | Did COVID-19 affect the total income or wages of your household? | 1. Decrease  2. Increase  0. No effect  -77. Refuse to answer |
|  | **To be asked only if Q 702=1**  List the reasons for the reduction in your household’s income?  [Multiple Response] | 1. Could not work due to travel/movement restrictions  2. Disruptions in markets (not able to sell products or buy inputs)  3. Lower prices for products we sell  4. Less customers/clients  5. Had to close shop/business  6. Household members sick or in quarantine  7. Loss of employment  8. Reduced salary/wage  9. Daily labour opportunities reduced  10. Support/assistance has been reduced  11. Less remittances  12. Pregnancy, childbirth, childcare  95. Other (specify)_____________ |
|  | What other types of difficulties did you or your household face due to COVID-19?  CAPI Instructions:  **Option 1 to appear only if respondent says 1 in Q702.**  *(Multiple response)* | 1. Unemployment/loss of income 2. Shortages in food supply 3. Shops being closed 4. Travel restrictions 5. Social distancing 6. Quarantine or self-quarantine 7. Being sick or fear of getting sick 8. Loss of HH member 9. Fear of dying   95. Others (specify) |
|  | Does your household have the following items? | 0. No  1. Yes |
|  | 1. Electricity |  |
|  | 1. Mattress |  |
|  | 1. Pressure cooker |  |
|  | 1. Chair |  |
|  | 1. Cot/Bed |  |
|  | 1. Table |  |
|  | 1. Electric fan |  |
|  | 1. Radio |  |
|  | 1. Television |  |
|  | 1. Sewing machine |  |
|  | 1. Mobile telephone |  |
|  | 1. Any other telephone |  |
|  | 1. Computer |  |
|  | 1. Refrigerator |  |
|  | 1. Watch/clock |  |
|  | 1. Bicycle |  |
|  | 1. Motorcycle/Scooter |  |
|  | 1. Animal drawn cart |  |
|  | 1. Car |  |
|  | 1. Water pump |  |
|  | 1. Thresher |  |
|  | 1. Tractor |  |

**MODULE H: COPING STRATEGIES**

**Read Out:** During the pastyear, did anyone in your household have to engage in any of the following behaviours due to a lack of food or a lack of money to buy food or meet other basic needs?

| **Q. No.** | **Question** | **Responses**  0. No  1. Yes  -99. Don’t know |
| --- | --- | --- |
|  | Spent savings |  |
|  | Reduced food consumption |  |
|  | Reduced health expenditure |  |
|  | Reduced other essential non-food expenditures such as education and clothes |  |
|  | Bought food on credit |  |
|  | Borrowed money to buy food |  |
|  | Reduced expenses on agricultural, livestock or fisheries inputs  (such as fertilizers, feed for fish/poultry/livestock, irrigation) |  |
|  | Sold jewellery/gold |  |
|  | Sold household goods (radio, furniture, mobile, solar panel, television, clothes, kitchen items, etc.) |  |
|  | Sold productive assets or means of transport  (cow/livestock, sewing machines, wheelbarrow, bicycle, livestock etc.) |  |
